# Clinical and Radiological Fusion: A New Frontier in Predicting Post-Transplant Diabetes Mellitus

**DOI:** 10.3389/ti.2025.14377

**Published:** 2025-04-03

**Authors:** Pooja Budhiraja, Byron H. Smith, Aleksandra Kukla, Timothy L. Kline, Panagiotis Korfiatis, Mark D. Stegall, Caroline C. Jadlowiec, Wisit Cheungpasitporn, Hani M. Wadei, Yogish C. Kudva, Salah Alajous, Suman S. Misra, Hay Me Me, Ian P. Rios, Harini A. Chakkera

**Affiliations:** ^1^ Department of Medicine, Mayo Clinic Arizona, Phoenix, AZ, United States; ^2^ Department of Quantitative Health Sciences, Mayo Clinic, Rochester, MN, United States; ^3^ Department of Medicine, Mayo Clinic, Rochester, MN, United States; ^4^ Department of Radiology, Mayo Clinic, Rochester, MN, United States; ^5^ Department of Surgery, Mayo Clinic, Rochester, MN, United States; ^6^ Department of Surgery, Mayo Clinic Arizona, Phoenix, AZ, United States; ^7^ Department of Transplant, Mayo Clinic Florida, Jacksonville, FL, United States

**Keywords:** post transplant diabetes, kidney transplant, obesity, adiposity, visceral diposity

## Abstract

This study developed a predictive model for Post-Transplant Diabetes Mellitus (PTDM) by integrating clinical and radiological data to identify at-risk kidney transplant recipients. In a retrospective analysis across three Mayo Clinic sites, clinical metrics were combined with deep learning analysis of pre-transplant CT images, focusing on body composition parameters like adipose tissue and muscle mass instead of BMI or other biomarkers. Among 2,005 nondiabetic kidney recipients, 335 (16.7%) developed PTDM within the first year. PTDM patients were older, had higher BMIs, elevated triglycerides, and were more likely to be male and non-White. They exhibited lower skeletal muscle area, greater visceral adipose tissue (VAT), more intermuscular fat, and higher subcutaneous fat (all p < 0.001). Multivariable analysis identified age (OR: 1.05, 95% CI: 1.03–1.08, p < 0.0001), family diabetes history (OR: 1.55, CI: 1.14–2.09, p = 0.0061), White race (OR: 0.43, CI: 0.28–0.66, p < 0.0001), and VAT area (OR: 1.37, CI: 1.14–1.64, p = 0.0009) as predictors. The combined model achieved C-statistic of 0.724 (CI: 0.692–0.757), outperforming the clinical-only model (C-statistic 0.68). Patients with PTDM in the first year had higher mortality than those without PTDM. This model improves predictive precision, enabling accurate identification and intervention for at risk patients.

## Graphical Abstract

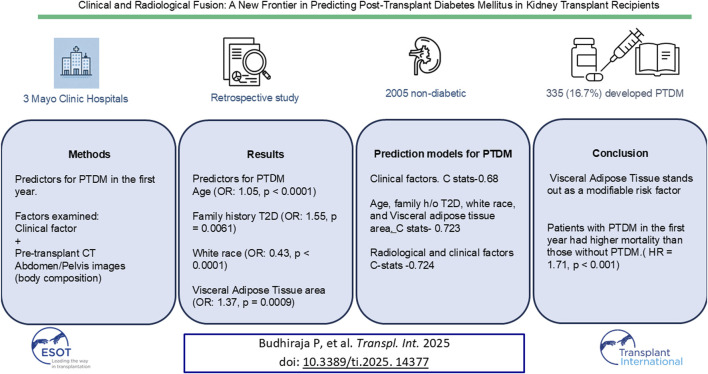

## Introduction

Post-transplant diabetes mellitus (PTDM) refers to the onset of diabetes in previously nondiabetic individuals following organ transplantation. The incidence of PTDM varies depending on the type of organ transplanted and the post-transplant period. Studies estimate that, at 12 months post-transplant, the incidence ranges from 10% to 30% for kidney transplant recipients [1–6]. This variation may be attributed to differences in diagnostic criteria for type 2 diabetes (T2D), diverse study populations, varying immunosuppression protocols, and the timeframes of the studies.

PTDM has a significant impact on transplant outcomes, being associated with an increased risk of graft rejection [7], infections [7], graft loss [8], cardiovascular mortality, and overall mortality [8–10]. In a United States Renal Data System study of 11,659 patients who received a kidney transplant between 1996 and 2000, PTDM was associated with a more than 60% increase in the incidence of graft failure and a 90% increase in mortality [10]. Additionally, PTDM negatively affects quality of life and substantially raises annual healthcare costs [11].

There is a nine-fold increased risk of diabetes in solid organ transplant recipients compared to their age-matched controls [12]. While the pathophysiology of PTDM mirrors that of T2D, it is further complicated by both transplantation-specific and non-transplantation-related risk factors [13]. The incidence of PTDM is rising, driven by the increasing number of kidney transplants, an aging recipient population, growing obesity trends, and the widespread use of tacrolimus [1, 9, 10, 12, 14].

Obesity is on the rise, leading to an increased risk of PTDM. Obesity is often assessed using body mass index (BMI), a widely used but limited measure [15–17].

BMI overlooks important variations in body composition and fat distribution across different ethnic groups, ages, and genders. It does not differentiate between muscle and fat mass, nor does it distinguish between subcutaneous and visceral adipose tissue (VAT)—the latter being more strongly associated with insulin resistance, metabolic syndrome, and elevated mortality [18, 19].

Given these limitations, there is increasing interest in using body composition analysis to provide deeper insights into metabolic health and improve the accuracy of PTDM risk prediction. Unlike BMI, a single axial computed tomography (CT) slice of the abdomen can visualize and quantify subcutaneous adipose tissue (SAT), visceral adipose tissue (VAT), intermuscular adipose tissue (IMAT), and skeletal muscle areas. These more detailed measurements provide a clearer understanding of PTDM risk factors and open new avenues for targeted interventions.

We propose a prediction model that incorporates body composition vs. BMI as a surrogate marker for obesity [20, 21]. Our team has developed a deep learning analysis of cross-sectional imaging to quantify body composition [22]. This algorithm automatically segments the following compartments: SAT, VAT, muscle, bone, and visceral organs. In the present study, we integrated clinical data with information from this deep learning model to predict PTDM.

Given the complexity and burden of PTDM, developing a comprehensive predictive tool incorporating body composition using deep learning can significantly enhance precision-based medicine for transplant recipients.

## Materials and Methods

Study Design and Setting: This is a retrospective study of the three Mayo Clinic sites. (Arizona, Florida, and Rochester). The Mayo Clinic Institutional Review Board approved this study.

Participants: The subjects were from three Mayo sites. The Mayo Clinic Arizona cohort was selected from 1/2007 to 1/2022, and the other two sites were included from 1/2014 to 1/2022 due to changes in the pre-transplant candidate imaging testing protocol. The cohort included both living and deceased donor transplants. The last follow-up was at the end of 1/2023.

Preoperative CT scans were primarily performed for vascular assessment, which has become the standard of care for evaluating kidney transplant candidates. Initially, CT imaging was selectively used in patients with peripheral vascular disease, diminished distal pulses, polycystic kidney disease, or a history of previous transplants. The primary purpose of these scans was to assess vascular anatomy and identify potential complications that could impact the surgical approach. Over time, as the clinical benefits of comprehensive vascular imaging became evident, preoperative CT scans were expanded to include all transplant candidates to ensure thorough pre-surgical planning and risk assessment. The study was approved by the Mayo institutional review board and was conducted in compliance with the Declaration of Helsinki.

Inclusion criteria:

• Kidney transplant recipients who have had:
  ○ Pre-operative CT abdomen and pelvis within 1 year before transplant or 1 month after kidney transplant
  ○ At least 1 year of follow-up at Mayo Clinic
  ○ Patient and graft surviving at 1 year.

Exclusion criteria:

• Patients with pre-existing Diabetes Mellitus (DM)
• Multivisceral organ transplants.
• Previous kidney transplant

### Immunosuppression Protocol

All patients received induction immunosuppression. Before 2011, patients received induction with rabbit‐anti thymocyte globulin. After 2011, induction was with Alemtuzumab. Patients over 65 received Basiliximab, which did not change during the study period. Patients receiving induction with the depleting agents had a complete withdrawal of corticosteroids by post-transplant day 5, while those receiving Basiliximab inductions continued maintenance corticosteroids. Steroids were maintained if they had panel reactive antibody >80%, donor-specific antibody, or end-stage renal disease from glomerulonephritis. Maintenance immunosuppression was with tacrolimus and mycophenolate mofetil. The trough tacrolimus levels were 8–10 ng/mL for the first month and then 6–8 ng/mL.

### Diagnosis of PTDM

In this study, we diagnosed PTDM using the American Diabetes Association definition based on Hba1c ≥6.5%, or fasting blood sugar ≥126 mg/dL, or random glucose ≥200 mg/dL or medications for diabetes management [23].

### Clinical Model for PTDM Prediction

In our previous work, we examined PTDM risk using the clinical factors [17] through two multivariable approaches [1]: a standard model that included continuous and discrete variables without categorization and [2] a dichotomized model, where variables were assigned binary values based on clinically relevant cut points. In the standard model, continuous variables (such as recipient age, baseline BMI, steroid use, triglycerides, pretransplant fasting glucose, and family history of type 2 diabetes) were included and weighted according to their β-coefficients in the multivariable logistic model. In the dichotomous model, continuous variables were dichotomized based on clinically relevant cut points (values below and above the cut point assigned 0 and 1, respectively) and weighted according to the β-coefficients. This approach included age ≥50 years, BMI ≥30 kg/m^2^, steroid use post-transplant, triglycerides ≥200 mg/dL, pretransplant fasting glucose ≥100 mg/dL, and family history of T2D.

Building on this foundation, we aimed to develop a more advanced and comprehensive predictive model for PTDM by integrating clinical and radiological data. This new model includes body composition measures derived from automated CT analysis, which provide a more precise assessment than BMI. We compared the performance of our previously established clinical model with this enhanced radiological approach, enabling a detailed assessment of PTDM risk linked to specific body composition profiles.

### Automated Body Composition Analysis

Mayo Clinic has previously developed deep learning models that automatically calculate highly accurate body composition measurements from CT images to inform individual care. These models use a fully automated abdominal segmentation deep neural network [22]. Furthermore, our model can segment SAT and VAT, muscle, abdominal organs, and bone; most fully automated algorithms are demonstrated on adipose tissue and muscle alone. We obtained the following measures: skeletal muscle, SAT, VAT, and IMAT.

Examinations were segmented into four compartments—subcutaneous adipose tissue, muscle, viscera, and bone—and pixels external to the body. The visceral compartment was further separated into VAT-free tissue and VAT using thresholding. Visceral adipose tissue-free tissue is primarily composed of abdominal organs, vessels, and the contents of the digestive tract. Further details of the model are available in the manuscript by Weston et al. [22].

To determine whether a model trained on a 2D section at the level of the Lumbar 3 transverse processes could generalize across the entire abdomen, L2 complete examinations of the abdomen from the inferior endplate of the L1 vertebra to the superior endplate of the L5 vertebra were used. Each section in this range was segmented. This is an example of a three-dimensional model using the deep learning algorithm developed by the group. The image variables were scaled by their standard deviation (using standardization).

### Statistical Analysis

Descriptive statistics were reported as mean (standard deviation) for continuous variables and frequency (percentage) for categorical variables. We compared continuous variables in 2 groups using a Student’s t-test and dichotomous outcomes using chi-square. Nonparametric tests compared heavily skewed data. A p-value <0.05 was considered statistically significant. Missing data was not imputed; models were only fit on complete datasets.

We analyzed factors associated with the development of PTDM using univariate analysis. The factors significant in univariate analysis were included in the Multivariable analysis.

These models included:

1. The previously established clinical continuous model (Age, BMI at baseline, steroid maintenance, pretransplant fasting glucose, pre-transplant fasting triglycerides (log-transformed), family history of T2DM [17].
2. Clinical discrete model (Age ≥50 years, BMI ≥30 kg/m^2^, steroid maintenance, fasting triglycerides ≥200 mg/dL, fasting glucose ≥100 mg/dL and family history of T2D) [17].
3. Baseline clinical factors that were significant in our model on univariate analysis
4. Model with radiology morphometric features (skeletal muscle area, SAT area, VAT area, IMAT area)
5. The model combining model 3 (Baseline factors that were significant in our model on the univariate analysis) and model 4 (radiology morphometric features)
6. Model with baseline factors that were significant on a multivariable analysis of model 5.

We evaluated the performance of various predictive models for diabetes mellitus post-transplant using the C-statistic. The comparisons were conducted on the same population to ensure consistency. The C-statistics and their corresponding 95% confidence intervals (CIs) were calculated, and cross-validation was performed to obtain mean C-statistics. Additionally, a C-statistic comparison was executed using the infinitesimal jackknife method.

We also examined the impact of the development of PTDM within the first year on patient and graft survival.

All statistical analyses were performed using the R Statistical Program, Version 4.2.2 (R Foundation for Statistical Computing, Vienna, Austria).

## Results

In a cohort of 2,005 nondiabetic kidney transplant recipients, PTDM occurred in 335 patients (16.7%) within the first year post-transplant. The mean age of recipients was 52.6 years (SD = 14.2), and 56.9% were male (Table 1).

**TABLE 1:** Demographics and clinical characteristics.

Variable	No PTDM (N = 1,670)	Developed PTDM (N = 335)	Total (N = 2005)	p-value
Sex Female Male	733 (44.5%) 916 (55.5%)	119 (36.2%) 210 (63.8%)	852 (43.1%) 1,126 (56.9%)	0.006
Age at Transplant	51.3 (14.2)	58.6 (12.4)	52.6 (14.2)	<0.001
RACE African American Asian Hispanic Native American Other White	203 (12.2%) 67 (4.0%) 192 (11.5%) 32 (1.9%) 45 (2.7%) 1,131 (67.7%)	58 (17.3%) 23 (6.9%) 42 (12.5%) 4 (1.2%) 11 (3.3%) 197 (58.8%)	261 (13.0%) 90 (4.5%) 234 (11.7%) 36 (1.8%) 56 (2.8%) 1,328 (66.2%)	0.008
Age of donor	40.3 (14.9)	42.6 (15.4)	40.7 (15.0)	0.011
Dialysis before transplant No Yes	447 (27.1%) 1,202 (72.9%)	94 (28.7%) 234 (71.3%)	541 (27.4%) 1,436 (72.6%)	0.565
Weight (kilogram)	75.9 (18.4)	80.1 (19.2)	76.6 (18.6)	<0.0001
Body mass index (kg/m^2^)	25.9 (5.1)	27.0 (5.4)	26.1 (5.2)	<0.0001
C-peptide before transplant Median (Interquartile range) (ng/mL)	7.200 (4.425, 11.175)	8.200 (4.900, 14.300)	7.300 (4.600, 11.700)	0.942

The average age at transplant was significantly higher for those who developed PTDM, at 58.6 years (SD = 12.4), compared to 51.3 years (SD = 14.2) for those who did not develop PTDM (p < 0.001). In the post-transplant diabetes mellitus (PTDM) group, 34.7% of patients received a living donor kidney transplant, compared to 39.4% in the non-PTDM group (p = 0.105). The proportion of male recipients was significantly higher among those who developed PTDM (63.8%) compared to those who did not (55.5%) (p = 0.006). The racial distribution also differed significantly between the two groups. Among patients who developed PTDM, 58.8% were White, 17.3% African American, 6.9% Asian, 12.5% Hispanic, 1.2% Native American, and 3.3% Other. In contrast, among those who did not develop PTDM, the distribution was 67.7% White, 12.2% African American, 4.0% Asian, 11.5% Hispanic, 1.9% Native American, and 2.7% Other (p = 0.008). This suggests that the proportion of White recipients was lower among those who developed PTDM (58.8% vs. 67.7%), while the proportions of African American, Asian, and Hispanic recipients were higher among those who developed PTDM.

The difference between the two groups in preemptive transplant versus dialysis status before transplant was not statistically significant (28.7% vs. 27.1%, p = 0.565). However, at baseline, PTDM patients had a significantly higher BMI (27.0 kg/m^2^ vs. 25.9 kg/m^2^, p < 0.001). C-peptide levels were similar between the groups.

### Radiological Characteristics

In this study, several key differences in body composition were observed between individuals who developed PTDM and those who did not (Table 2).

**TABLE 2:** Radiological factors.

Variable	No PTDM (N = 1,670)	Developed PTDM (N = 335)	Total (N = 2005)	Values-value
Skeletal muscle area				
Mean (SD)	3.7 (1.0)	3.9 (1.0)	3.7 (1.0)	0.001
Median (Q1, Q3):	3.6 (2.9, 4.4)	3.9 (3.2, 4.5)	3.6 (2.9, 4.4)	
Range	0.5–7.4	1.8–7.5	0.5–7.5	
Skeletal muscle mean HU				
Mean (SD)	3.3 (1.0)	3.0 (1.0)	3.2 (1.0)	<0.001
Median (Q1, Q3):	3.3 (2.6, 3.9)	2.9 (2.3, 3.7)	3.2 (2.6, 3.9)	
Range	−0.5–8.6	0.5–5.6	−0.5–8.6	
Subcutaneous adipose tissue area				
Mean (SD)	1.7 (1.0)	2.0 (1.0)	1.8 (1.0)	<0.001
Median (Q1, Q3):	1.6 (1.0, 2.3)	1.8 (1.2, 2.6)	1.6 (1.1, 2.3)	
Range	0.1–5.8	0.3–5.2	0.1–5.8	
Subcutaneous adipose tissue mean HU				
Mean (SD)	−4.0 (1.0)	−4.1 (0.9)	−4.0 (1.0)	0.084
Median (Q1, Q3):	−4.3 (−4.7, −3.7)	−4.3 (−4.7, −3.9)	−4.3 (−4.7, −3.7)	
Range	−5.6–7.2	−5.4–0.3	−5.6–7.2	
Visceral adipose tissue area				
Mean (SD)	1.3 (1.0)	1.8 (1.1)	1.3 (1.0)	<0.001
Median (Q1, Q3):	1.1 (0.5, 1.8)	1.7 (1.0, 2.5)	1.2 (0.5, 1.9)	
Range	0.0–6.0	0.0–6.0	0.0–6.0	
Visceral adipose tissue area quartile Q1-3: Q4:	1,104 (78.6%) 301 (21.4%)	170 (57.8%) 124 (42.2%)	1,274 (75.0%) 425 (25.0%)	<0.001
Visceral adipose tissue mean HU				
Mean (SD)	−6.3 (1.0)	−6.6 (1.0)	−6.3 (1.0)	<0.001
Median (Q1, Q3):	−6.4 (−7.0, −5.7)	−6.8 (−7.3, −6.0)	−6.5 (−7.0, −5.7)	
Range	−17.0–3.1	−17.0–3.1	−17.0–3.1	
Intermuscular adipose tissue area				
Mean (SD)	1.7 (1.0)	2.1 (1.0)	1.8 (1.0)	<0.001
Median (Q1, Q3):	1.6 (1.1, 2.2)	1.9 (1.4, 2.4)	1.7 (1.1, 2.3)	
Range	0.0–11.3	0.3–8.9	0.0–11.3	
Intermuscular adipose tissue mean HU				
Mean (SD)	−13.8 (1.0)	−13.9 (0.9)	−13.8 (1.0)	0.318
Median (Q1, Q3):	−13.8 (−14.4, −13.2)	−13.9 (−14.3, −13.3)	−13.8 (−14.4, −13.2)	
Range	−17.3–9.3	−16.7–10.6	−17.3–9.3	

HU, Hounsfield units; SD, standard deviation; Q1, Quartile 1; Q3, Quartile 3.

PTDM patients had a lower skeletal muscle area (165.3 cm^2^ vs. 171.5 cm^2^, p = 0.001) and lower skeletal muscle mean Hounsfield Units (HU) (32.6 vs. 33.0, p = 0.001), indicating reduced muscle mass and poorer muscle quality compared to non-PTDM patients. HU values measure tissue density, and lower values indicate less healthy muscle.

Moreover, PTDM patients exhibited larger areas of both SAT (285.1 cm^2^ vs. 275.8 cm^2^, p = 0.001) and VAT (121.7 cm^2^ vs. 111.8 cm^2^, p = 0.001). Additionally, a higher proportion of PTDM patients (57.6%) were in the highest quartile (Q4) of VAT compared to non-PTDM patients (73.5% in Q1-Q3, p = 0.001).

There was also a significant increase in IMAT in PTDM patients (2.4 cm^2^ vs. 1.7 cm^2^, p = 0.001). This increase in IMAT is associated with reduced muscle function and metabolic health. No significant differences were found in the quality (HU values) of SAT (p = 0.084) or IMAT (p = 0.318). However, PTDM patients had slightly lower VAT HU values (−6.6 vs. −6.4, p = 0.001), suggesting that the visceral fat in PTDM patients was denser, which could be metabolically more harmful, as denser fat is associated with worse metabolic outcomes.

### Risk Factors for PTDM

In multivariable analysis, key predictors of PTDM included recipient age (OR: 1.05, 95% CI: 1.03–1.08, p < 0.0001), family history of type 2 diabetes (OR: 1.55, 95% CI: 1.14–2.09, p = 0.0061), White race (OR: 0.43, 95% CI: 0.28–0.66, p < 0.0001), visceral adipose tissue (VAT) area (OR: 1.37, 95% CI: 1.14–1.64, p = 0.0009), and weight change (OR: 1.02, 95% CI: 1.00–1.03, p = 0.013). Other factors, such as BMI, steroid use, and various adipose tissue measures, showed associations in univariate analysis but did not retain significance in the multivariable model (Table 3).

**TABLE 3:** Factors associated with the development of Post Transplant Diabetes Mellitus.

Variables	N univariate	Odds ratio (CI) univariate	P value univariate	N multivariable	Odds ratio (CI) multivariable	P value multivariable
Recipient Age	2005	1.04 (1.03, 1.05)	<0.0001	1,603	1.05 (1.03, 1.08)	<0.0001
BMI (Baseline)	1670	1.04 (1.02, 1.06)	0.001	1,603	1.03 (0.98, 1.07)	0.2110
Dialysis Duration	1977	0.93 (0.71, 1.21)	0.565	NA	NA	NA
Steroid maintenance	1968	1.38 (1.06, 1.82)	0.0180	1,603	1.27 (0.91, 1.80)	0.1669
Family h/o type 2 diabetes mellitus	1947	1.55 (1.20, 1.99)	0.0007	1,603	1.55 (1.14, 2.09)	0.0061
Triglyceride pre transplant	1213	1.00 (1.00, 1.00)	0.0382		1.00 (1.00, 1.00)	0.3640
Fasting glucose pre transplant	1115	1.01 (0.99,1.03)	0.4010	NA		
Sex Male	1978	1.41 (1.11, 1.81)	0.0058	1,603	1.05 (0.68, 1.64)	0.8137
Fasting glucose	1644	0.98 (0.86, 1.12)	0.8041	NA	NA	NA
Asian	2005	1.20 (0.68, 2.08)	0.5178	1,603	1.01 (0.49, 2.01)	0.9753
Hispanic	2005	0.77 (0.49, 1.19)	0.2378	1,603	0.63 (0.35, 1.12)	0.1168
Native American	2005	0.44 (0.13, 1.16)	0.1334	1,603	0.29 (0.06, 0.94)	0.0629
Other Race	2005	0.86 (0.40, 1.71)	0.6715	1,603	0.73 (0.22, 2.02)	0.5696
White	2005	0.61 (0.44, 0.85)	0.0032	1,603	0.43 (0.28, 0.66)	<0.0001
Skeletal Muscle Area	1699	1.23 (1.09, 1.39)	0.0011	1,603	1.06 (0.84, 1.33)	0.6311
Subcutaneous Adipose Tissue Area	1699	1.26 (1.12, 1.42)	<0.0001	1,603	1.02 (0.81, 1.29)	0.8390
Intermuscular Adipose Tissue Area	1699	1.35 (1.20, 1.51)	<0.0001	1,603	0.97 (0.81, 1.15)	0.7578
Visceral Adipose Tissue Area	1699	1.63 (1.45, 1.84)	<0.0001	1,603	1.37 (1.14, 1.64)	0.0009

BMI, Body mass index; CI, Confidence Interval.

### Models for PTDM Prediction

Table 4 summarizes the results of various predictive models for PTDM. The previously established clinical continuous model in this study achieved a C-statistic of 0.68 (95% CI: 0.636, 0.724) with a mean cross-validated C-statistic of 0.676. The clinical discrete model, which used binary cut-points for key variables, had a C-statistic of 0.656 (95% CI: 0.612, 0.699) and a mean cross-validated C-statistic of 0.651 (Table 4).

**TABLE 4:** Models for post-transplant diabetes mellitus prediction.

Model	Variables	C-statistic	Mean Cross-validated C-statistic
Continuous model	Recipient Age, baseline Body mass index, steroid maintenance, pretransplant fasting glucose, pre-transplant fasting triglycerides (log-transformed), family history of diabetes mellitus	0.68 (0.636, 0.724)	0.676
Discrete model	Age ≥50, baseline Body mass index ≥30 kg/m^2^, steroid maintenance, pretransplant fasting glucose ≥100 mg/dL fasting triglycerides ≥200 mg/dL, family history of diabetes mellitus	0.656 (0.612, 0.699)	0.651
Baseline clinical factors significant on univariate analysis	Sex, age, race, baseline Body mass index, family history of diabetes	0.701 (0.668, 0.734)	0.686
Radiology only	Skeletal muscle area, subcutaneous adipose tissue area, visceral adipose tissue area, intermuscular adipose tissue area	0.658 (0.625, 0.692)	0.656
Baseline factors significant on univariate analysis with radiology (baseline + radiology)	Sex, age, race, baseline Body mass index, family history of diabetes mellitus, skeletal muscle area, subcutaneous adipose tissue area, visceral tissue area, intermuscular adipose tissue area	0.724 (0.692, 0.757)	0.705
Baseline Variables significant on multivariable analysis	Age, family history of diabetes mellitus, race, visceral adipose tissue area	0.723 (0.691, 0.754	0.714

Baseline clinical factors that were significant on univariate analysis (sex, recipient age, race, baseline BMI, and family history of type 2 diabetes) achieved a C-statistic of 0.701 (95% CI: 0.668, 0.734) with a mean cross-validated C-statistic of 0.686. When these baseline clinical factors were combined with radiological measures (skeletal muscle area, SAT area, VAT area, and IMAT area), the “Baseline + radiology” model achieved the highest C-statistic of 0.724 (95% CI: 0.692, 0.757) and a mean cross-validated C-statistic of 0.705. This finding suggests that integrating radiological factors with clinical data yields the most accurate prediction of PTDM risk in this study.

The multivariable significant variables model, which included only age, family history of diabetes, race, and VAT, demonstrated nearly equivalent predictive performance with a mean cross-validated C-statistic of 0.714. This streamlined model provides a strong balance of predictive accuracy and simplicity, making it potentially more practical for clinical application.

### Survival Analysis

**FIGURE 1:**
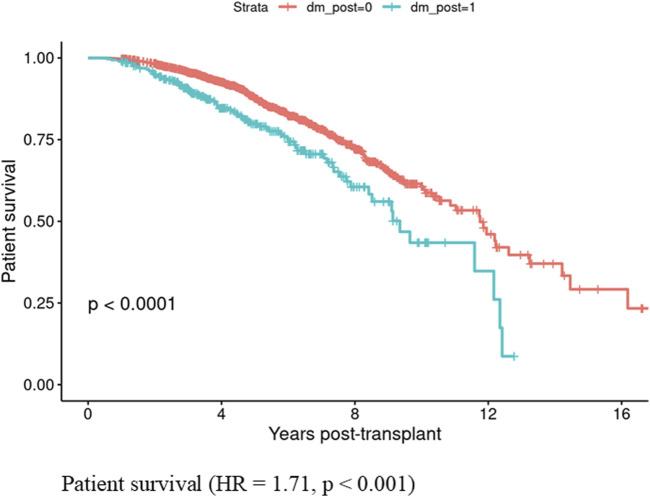
Patient survival in those who developed Posttransplant diabetes mellitus vs., those who did not did not develop posttransplant diabetes mellitus in the first year if kidney transplant.

**FIGURE 2:**
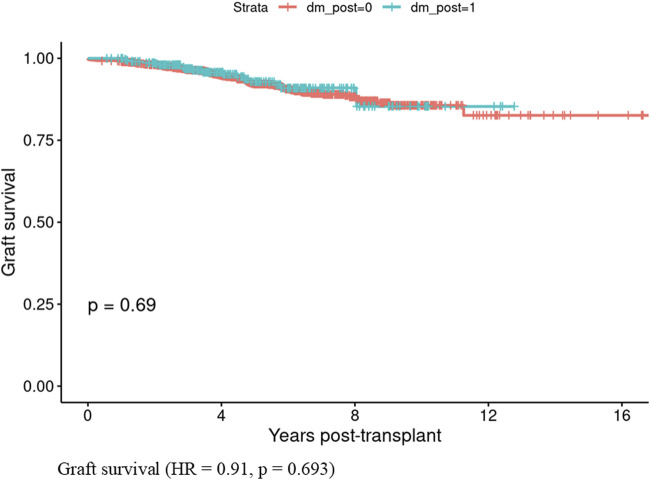
Graft survival in those who developed Posttransplant diabetes mellitus vs., those who did not develop posttransplant diabetes mellitus in the first year of kidney transplant.

Patients who developed PTDM within the first year showed lower patient survival rates compared to those who did not develop PTDM (HR = 1.71, CI: 1.33–2.21, p < 0.001) (Figure 1). In contrast, graft survival in the first year was comparable between patients with and without PTDM (HR = 0.91, CI 0.56–1.47, p = 0.693) (Figure 2).

## Discussion

This study presents a comprehensive model for predicting PTDM in kidney transplant recipients, utilizing both clinical and advanced radiological data. Our model is innovative in incorporating body composition, moving beyond the conventional BMI-based obesity assessment, and providing a higher precision to identify high-risk PTDM patients preemptively. In a large, diverse cohort of 2,005 nondiabetic kidney transplant recipients, 335 (16.7%) developed PTDM within the first year. Older age, family history of diabetes, nonwhite race, and increased VAT are significant predictors of PTDM. Importantly, patients with PTDM within the first year post-transplant demonstrated significantly higher mortality (HR = 1.71, p < 0.001) compared to those without PTDM, highlighting the adverse impact of PTDM on patient longevity.

Our model achieved high predictive performance, with the combination of baseline clinical factors and radiological measures (“Baseline + radiology” model) reaching a C-statistic of 0.724 (95% CI: 0.692, 0.757), surpassing traditional clinical models with a C-statistic of 0.68. This improvement highlights the value of integrating radiological factors, particularly VAT, with clinical data to enhance PTDM prediction accuracy. A simplified model with variables from multivariable analysis (age, family history of diabetes, race, and VAT) achieved similar predictive value with a C-statistic of 0.723 (95% CI: 0.691, 0.754) and a cross-validated C-statistic of 0.714, suggesting this precise model offers clinical practicality without compromising accuracy.

Among the predictors identified, VAT stands out as a modifiable risk factor, while age, family history of T2D, and race is nonmodifiable. Our findings underscore VAT’s role as a stronger predictor of PTDM than BMI. Patients who developed PTDM had significantly larger VAT areas (121.7 cm^2^ vs. 111.8 cm^2^), more intramuscular fat (2.4 cm^2^ vs. 1.7 cm^2^), and lower skeletal muscle mass (165.3 cm^2^ vs. 171.5 cm^2^), indicating the critical impact of fat distribution and muscle quality on PTDM risk. Increased VAT intramuscular fat and reduced muscle mass may impair glucose metabolism, promoting insulin resistance and PTDM development [18, 19].

PTDM patients exhibited higher subcutaneous and visceral fat levels, particularly a significantly larger VAT area. VAT is strongly associated with metabolic risks, including diabetes, and is considered more harmful than SAT due to its location, secretions, and contribution to insulin resistance. While previous models relied on BMI, triglycerides, HDL, uric acid, and fasting glucose markers to assess metabolic risk, these markers do not fully capture the underlying metabolic dysfunction. In contrast, VAT, as a metabolically active tissue, plays a key role in insulin resistance and metabolic dysregulation, which was strongly supported by our findings where BMI was not significant, but VAT emerged as a robust predictor. VAT contains more immune cells than SAT, secreting higher levels of pro-inflammatory mediators and cytokines that exacerbate insulin resistance, contributing to diabetes [18, 19]. This unique secretome of VAT has a distinct and negative impact on hepatocyte and muscle insulin action, highlighting the depot-specific differences in adipose tissue secretome composition and their effects on metabolic syndrome and diabetes. By incorporating VAT as a central feature, our model provides a more precise reflection of metabolic risks compared to the previous reliance on traditional markers.

While Ji Eun Kim et al. [24] used deep learning-based quantification of 3D visceral fat volume, their study focused solely on body composition analysis for PTDM without integrating clinical risk factors into a predictive model. Their approach was based on volumetric analysis of total visceral fat. In contrast, our study incorporates CT-derived VAT area measurements combined with clinical parameters to develop a comprehensive predictive model for PTDM. This distinction enhances the practical applicability of our model in transplant decision-making, allowing for better risk stratification and clinical translation than a body composition-only approach.

Furthermore, Feng et al. [25] identified intermuscular adipose tissue (IMAT) as the primary driver of PTDM, while our study found VAT to be the strongest predictor. These discrepancies likely arise from differences in study populations, imaging methodologies, and statistical models. Importantly, IMAT was significant in univariate analysis but did not remain significant in the multivariable model. In contrast, VAT remained an independent predictor of PTDM along with age, race, and family history of diabetes. This suggests that IMAT’s effect was confounded by stronger predictors, particularly VAT, which has a well-established role in insulin resistance and metabolic dysfunction. Given VAT’s pro-inflammatory profile, direct portal exposure, and stronger association with metabolic syndrome, its predictive value surpassed that of IMAT.

Unlike prior studies, which were often constrained by small sample sizes and a lack of diverse populations, our study stands out due to its large, multicenter design and incorporation of advanced radiological analysis [24–26]. Future research should evaluate how different fat depots contribute to PTDM risk in various transplant populations and explore whether a combined VAT + IMAT model could further enhance predictive accuracy. Additionally, we acknowledge the potential value of impedance-based techniques, such as multi-frequency BIA and phase angle analysis, as alternative tools for assessing metabolic risk when CT imaging is unavailable.

Our findings highlight the VAT’s predictive value for PTDM. Unlike prior studies, which were often constrained by small sample sizes and a lack of diverse populations, our study stands out due to its large, multicenter design and the incorporation of advanced radiological analysis [24, 26]. By integrating age, family history of diabetes, race, and VAT area into our model, we achieved a high AUC for PTDM prediction, demonstrating the model’s robustness and clinical utility. Furthermore, deep learning-based body composition analysis provided precise and detailed insights into the relationship between VAT and PTDM risk, offering a more nuanced understanding than traditional approaches.

Given VAT’s modifiable nature, targeted interventions focused on VAT reduction—such as diet, exercise, and Glucagon-Like Peptide-1 receptor agonists (GLP-1 RAs)—could be promising. Studies have shown that GLP-1 RAs significantly decrease VAT content compared to other medications, placebos, and lifestyle interventions [27, 28]. Real-time Polymerase Chain Reaction and immunofluorescence studies show that GLP-1 receptors are more abundant in VAT and epicardial adipose tissue than in SAT [29]. Animal studies with liraglutide, a GLP-1RA, demonstrated a reduction in VAT and an increase in SAT, likely due to altered lipid metabolism [30]. Additionally, rodent models suggest that GLP-1 receptor activation enhances sympathetic activity, promoting VAT lipolysis over SAT. These medications effectively reduce VAT compared to other treatments [31, 32].

Metabolic risk factors pre-transplant may worsen after transplant. Our analysis also reveals that patients who developed PTDM exhibit worsened metabolic parameters, including elevated triglycerides, reduced HDL, and increased BMI post-transplant (Supplementary Table S1). Therefore reinforcing the need for early intervention pretransplant.

Advancing the research in the field of our study with a large cohort of over 2,000 diverse participants from three different sites provides greater reliability and generalizability compared to other smaller studies. Using deep learning to analyze CT scans, we achieved precise measurements of VAT, SAT, and muscle mass, offering better insights into PTDM risk than BMI and other clinic laboratory factors as surrogates for obesity. The identification of VAT as a key predictor of PTDM underscores the need for CT-based body composition analysis in pre-transplant evaluations, as BMI may miss high-risk individuals. Targeting VAT reduction through lifestyle changes, GLP-1 receptor agonists, or metabolic surgery could lower PTDM risk.

While our model is comprehensive, it has limitations. It is a retrospective study, and though we adjusted for key confounders, unmeasured variables could still impact results, and CT scans may not be widely done. Although CT-based VAT quantification offers a superior metabolic risk assessment compared to BMI and traditional clinical markers, CT imaging is not universally performed for all kidney transplant candidates. However, given that many centers already conduct preoperative CT scans for vascular and anatomical evaluation, leveraging these existing images for VAT analysis adds clinical value without additional radiation exposure or cost. In settings where CT is not routinely available, alternative methods such as Dual-Energy X-ray Absorptiometry (DXA) or bioelectrical impedance analysis (BIA) could be explored in future studies as potential surrogates for VAT estimation.

Second, while BMI, bioelectrical impedance analysis (BIA), and DXA are widely used for body composition assessment, they lack the ability to precisely differentiate visceral adipose tissue (VAT) from subcutaneous fat (SAT), which is crucial since VAT is the primary driver of insulin resistance and PTDM. Unlike BMI, which does not account for fat distribution, and BIA, which is influenced by hydration status, CT directly quantifies VAT, allowing for a more accurate assessment of metabolic risk. Skinfold calipers, though simple and inexpensive, only estimate subcutaneous fat and are operator-dependent, making them unreliable for deep fat compartments such as VAT.

Future research should assess whether emerging impedance-based technologies, such as multi-frequency BIA and phase angle analysis, can enhance metabolic risk prediction in transplant candidates. Third, our study excluded patients with previous kidney transplants to maintain a homogeneous study population and improve model validity. However, this exclusion may limit the generalizability of our findings to patients undergoing repeat transplantation, who often have different metabolic profiles and long-term immunosuppression exposure. Lastly, while the combined model incorporating clinical and radiological data improved predictive performance (C-statistic of 0.724 vs. 0.686 for clinical-only models), the absolute increase is moderate. However, even small gains in predictive accuracy are clinically relevant as they allow for earlier identification of high-risk patients, targeted lifestyle interventions, and personalized metabolic management strategies to mitigate PTDM risk. The most parsimonious model—incorporating only age, family history of diabetes, race, and VAT area—achieved a C-statistic of 0.723, reinforcing VAT’s independent predictive value. Unlike BMI, which does not account for fat distribution, VAT directly contributes to insulin resistance and systemic inflammation. Given that VAT is modifiable, identifying high-VAT patients early enables targeted lifestyle interventions, glucose monitoring, and adjustments to immunosuppressive regimens.

Thus, while the numerical increase in C-statistic may seem moderate, its clinical implications are substantial, reinforcing VAT’s value in pre-transplant metabolic risk assessment. Future research should explore machine learning-based models and additional metabolic biomarkers to further refine PTDM prediction.

This precise model provides a valuable conceptual framework for stratifying risk, continuing efforts to adopt it into mainstream practice, and guiding targeted therapies for high-risk patients.

This study highlights the importance of body composition, particularly VAT and muscle mass, in predicting PTDM risk among kidney transplant recipients. By integrating clinical factors with radiological metrics, our model demonstrated greater predictive accuracy than traditional BMI-based assessments, emphasizing the need for CT-based body composition analysis in pre-transplant evaluations. While factors like age, family history of diabetes, and race are nonmodifiable, VAT represents a valuable modifiable target for intervention. Our findings also indicate that metabolic risks often worsen post-transplant, suggesting that transplantation alone does not fully address these challenges.

Our model provides a more sensitive identification of high-risk patients identified before or shortly after transplantation. Future research is needed to validate this model across different populations and healthcare settings to ensure broader applicability. Additionally, studies should explore timely interventions aimed at VAT reduction and muscle preservation—such as lifestyle modifications, pharmacologic agents like GLP-1 receptor agonists, or bariatric surgery—to maximize the benefits of these strategies. Early, tailored interventions could reduce PTDM incidence, improve patient survival, and enhance graft outcomes. This comprehensive model lays the framework for precision medicine, enabling early identification of at-risk individuals for PTDM and optimizing post-transplant care.

## Data Availability

Data is available on request due to privacy/ethical restrictions. The data that support the findings of this study are available on request from the corresponding author. The data are not publicly available due to containing research participant information.

## Abbreviations

AUC, Area under the curve; BMI, Body Mass Index; CI, Confidence intervals; CT, Computed Tomography; DM, Diabetes Mellitus; DXA, Dual-Energy X-ray Absorptiometry; GLP-1 RAs, Glucagon-Like Peptide-1 receptor agonists; IMAT, Intermuscular adipose tissue; PTDM, Post Transplant Diabetes Mellitus; SAT, Subcutaneous adipose tissue; T2D, Type 2 diabetes; VAT, Visceral adipose tissue.
