# Supplementary material for: Clinical and Radiological Fusion: A New Frontier in Predicting Post-Transplant Diabetes Mellitus
Source: Transpl Int. 2025 Apr 3;38:14377. doi: 10.3389/ti.2025.14377 (PMC12003133; doi:10.3389/ti.2025.14377)
Supplement: Supplementary file 1 [file Table1.DOCX]

Supplementary table S1

| **Variable** | **No PTDM (N=1670)** | **Developed PTDM (N=335)** | **Total (N=2005)** | **p-value** |
| --- | --- | --- | --- | --- |
| **Triglyceride at Transplant (mg/dl)** | 148.14 (90.91) | 162.82 (103.4) | 150.71 (93.35) | 0.037 |
| **Triglyceride at 4 months (mg/dl)** | 168.51 (105.67) | 184.11 (108.34) | 171.15 (106.25) | 0.023 |
| **Triglyceride at 8 months (mg/dl)** | 170.48 (102.73) | 220.31 (156.17) | 178.68 (114.75) | <0.001 |
| **Triglyceride at 12 months (mg/dl)** | 167.6 (106.36) | 206.93 (179.86) | 173.63 (121.33) | <0.001 |
| **HDL at 1 month (mg/dl)** | 54.5 (18.53) | 52.4 (19.14) | 54.1 (18.64) | 0.081 |
| **HDL at 4 months (mg/dl)** | 54.5 (18.64) | 50.7 (16.23) | 53.9 (18.31) | 0.001 |
| **HDL at 8 months (mg/dl)** | 52.7 (17.96) | 47.0 (15.82) | 51.8 (17.75) | 0.003 |
| **HDL at 12 months (mg/dl)** | 54.0 (18.61) | 49.1 (16.03) | 53.2 (18.31) | <0.001 |
| **Weight at baseline (kg)** | 75.9 (18.4) | 80.1 (19.2) | 76.6 (18.6) | <0.0001 |
| **Weight at 1 month (kg)** | 78.6 (19.3) | 86.1 (20.4) | 79.8 (19.7) | <0.001 |
| **Weight at 4 months (kg)** | 76.8 (18.9) | 82.8 (20.7) | 77.8 (19.3) | <0.001 |
| **Weight at 8 months (kg)** | 78.6 (19.1) | 85.4 (21.1) | 79.7 (19.6) | <0.001 |
| **Weight at 12 months (kg)** | 80.3 (19.7) | 87.6 (21.5) | 81.5 (20.2) | <0.001 |
| **BMI at baseline (kg/m²)** | 25.9 (5.1) | 27.0 (5.4) | 26.1 (5.2) | <0.0001 |
| **BMI at 1 month (kg/m²)** | 27.1 (6.8) | 29.2 (6.0) | 27.4 (6.7) | <0.001 |
| **BMI at 4 months (kg/m²)** | 26.6 (8.9) | 28.1 (6.2) | 26.9 (8.5) | 0.004 |
| **BMI at 8 months (kg/m²)** | 27.2 (8.9) | 29.0 (6.2) | 27.5 (8.5) | <0.001 |
| **BMI at 12 months (kg/m²)** | 27.6 (5.7) | 29.8 (6.4) | 28.0 (5.8) | <0.001 |

The above values for continuous values as Mean (standard deviation)

BMI: Body mass index

HDL: High-Density Lipoprotein
